# Comparative Morphological, Physiological, and Transcriptomic Analyses of Diploid and Tetraploid Wucai (*Brassica campestris* L.)

**DOI:** 10.3390/plants13162341

**Published:** 2024-08-22

**Authors:** Jian Wang, Ruxi Wang, Fan Luo, Wenjing Du, Jinfeng Hou, Guohu Chen, Xiaoyan Tang, Jianqiang Wu, Wenjie Wang, Bin Huang, Chenggang Wang, Lingyun Yuan

**Affiliations:** 1 Vegetable Genetics and Breeding Laboratory, College of Horticulture, Anhui Agricultural University, Hefei 230036, China; wangjian@stu.ahau.edu.cn (J.W.); wrx1207@stu.ahau.edu.cn (R.W.); ffanluo@outlook.com (F.L.); 23720173@stu.ahau.edu.cn (W.D.); houjinfeng@ahau.edu.cn (J.H.); cgh@ahau.edu.cn (G.C.); txy@ahau.edu.cn (X.T.); wujq@ahau.edu.cn (J.W.); wenjie_wang@ahau.edu.cn (W.W.); huangbin1123@outlook.com (B.H.); 2 Anhui Provincial Engineering Laboratory of Horticultural Crop Breeding, Hefei 230036, China; 3 Department of Vegetable Culture and Breeding, Wanjiang Vegetable Industrial Technology Institute, Maanshan 238200, China

**Keywords:** *Brassica campestris*, autotetraploid, development, transcriptome, glycolysis

## Abstract

Polyploid plants often exhibit superior yield, stress resistance, and quality. In this study, homologous tetraploid wucai (*Brassica campestris* L.) was successfully obtained by spraying seedling growth points with colchicine. The morphological, cytological, and physiological characteristics of diploid and tetraploid wucai were analyzed, and transcriptomic sequencing was performed at three stages of development. Tetraploid seedings grew slowly but exhibited darker leaves, enlarged organs and cells, increased stomatal volume, decreased stomatal density, improved nutritional content, and enhanced photosynthesis. Differentially expressed genes (DEGs) identified in diploid and tetraploid plants at three stages of development were enriched in different pathways. Notably, DEGs identified in the tetraploid plants were specifically enriched in starch and sucrose metabolism, pentose and glucuronate interconversions, and ascorbate and aldarate metabolism. In addition, we found that the light green module was most relevant to ploidy, and DEGs in this module were significantly enriched in the glycolysis/gluconeogenesis and TCA cycle pathways. The differential expression of key glycolysis-associated genes at different developmental stages may be the driver of the observed differences between diploid and tetraploid wucai. This study lays a technical foundation for the development of polyploid wucai germplasm resources as well as the breeding of new varieties with improved quality, yield, and stress resistance. It also provides a good empirical reference for the genetic breeding of closely related *Brassica* species.

## 1. Introduction

Polyploidization, or whole genome duplication (WGD), is considered an important mechanism in plant adaptation to environmental change [1]. Polyploidization is thought to be an evolutionary force in plants and animals, including invertebrates, fish, and amphibians [2,3,4]. Research suggests that the majority of angiosperms have experienced at least one WGD event during their evolutionary history [5]. Nearly 70% of angiosperms are polyploids [6], including many important crops such as polyploid potatoes [7] and bananas [8] and allopolyploid wheat [9] and cotton [10]. Polyploidization results in not only an increase in chromosome number but also additional genomic interactions and genetic alterations, making this process a reliable technique for crop improvement. For example, polyploidization has been successfully implemented in crop breeding programs to increase total yield and biomass production, including in sugar beets and watermelons [11].

Phenotypic variation resulting from polyploidization can contribute to agricultural productivity and efficiency, most notably by impacting the morphology and physiology of offspring. For example, polyploids often have larger cells than corresponding diploids, resulting in the enlargement of plant organs such as leaves, flowers, and seeds [12]. Polyploidization can also result in reduced height, thickened leaves, deepened leaf color, increased stomatal volume, and decreased stomatal density [13]. These variations in leaf characteristics can alter the photosynthetic performance of tetraploid plants. For instance, tetraploid barley has larger, thicker leaves containing a higher concentration of photosynthetic pigments, resulting in a significantly higher net photosynthetic rate and increased photosynthetic capacity under strong light [14]. In addition, tetraploid *Elsholtzia splendens* has a significant biomass advantage over diploid plants. Biomass accumulation is synergistically regulated by the photosynthetic chlorophyll a/b-binding protein, chloroplast synthesis, photosynthetic electron transfer, photophosphorylation, carbon assimilation, transcription factors, and endogenous phytohormones [15].

Polyploid plants exhibit an increase in the number of gene copies and alterations in gene expression regulation, resulting in the emergence of novel phenotypes [16]. In diploid and tetraploid col-0 and ler-0 *Arabidopsis thaliana*, genes regulated by ploidy level exhibit tissue specificity and are closely related to genotype [16]. In rice, the endosperm tissue contains a greater number of differentially expressed genes (DEGs) induced by homologous polyploidization than the leaves [17]. In addition, research suggests that DEGs induced by polyploidization tend to be related to stress response pathways. Homologous polyploidization may therefore enhance plant adaptability [18].

Wucai (*Brassica campestris* L. ssp. *chinensis* var. *rosularis* Tsen) is an important non-heading Chinese cabbage widely planted in the Chinese Yangtze–Huaihe Valley, suitable for open field cultivation in autumn and winter. However, ploidy breeding, an effective method of creating novel germplasm resources, has not yet been applied to wucai. In this study, we induced autotetraploidy in wucai by treating seedlings with colchicine. We further evaluated the phenotypic and cytological differences between diploid and tetraploid wucai and resolved the molecular mechanism responsible for the observed variations through transcriptomic sequencing.

## 2. Results

### 2.1. Identification of Diploid and Tetraploid Wucai Lines

**Figure 1:**
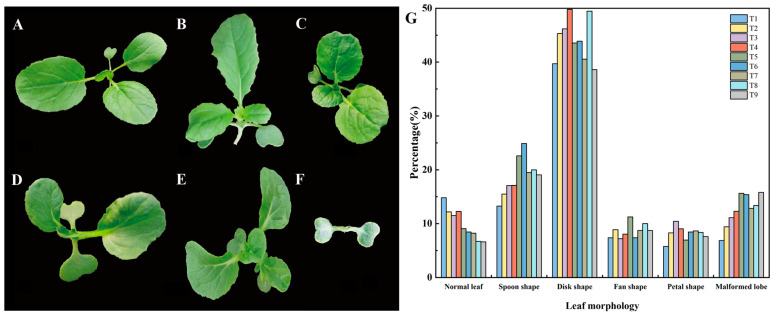
Morphological variation among wucai leaves following colchicine treatment. (**A**) Normal leaf; (**B**) Spoon shape; (**C**) Disk shape; (**D**) Fan shape; (**E**) Petal shape; (**F**) Malformed lobe; (**G**) Statistics of morphological variation.

Diploid wucai ‘W7-2’ cotyledons were treated with different concentrations of colchicine (0.1%, 0.2%, 0.3%) and different numbers of treatments (2, 4, 6) to induce autotetraploidy. Overall, the mutation rate increased and survival rate decreased with the increasing colchicine concentration and number of treatments. The highest tetraploid rate and doubling rate were observed after six treatments with 0.2% colchicine, resulting in nine tetraploid plants with a doubling rate of 4.76%. The lowest survival rate, variation rate, and doubling rate were observed after six treatments with 0.3% colchicine, as higher concentrations of colchicine were more toxic and hindered plant growth (Table 1). Following colchicine treatment, the leaves of diploid wucai exhibited significant morphological variation. In total, five distinct morphological variations were observed compared to normal leaves, with disc-shaped leaves being the most common, followed by fan- and petal-shaped leaves (Figure 1). Plants with deformed leaves were unable to grow and develop normally due to necrotic growth points.

### 2.2. Identification of Autotetraploid Wucai

**Figure 2:**
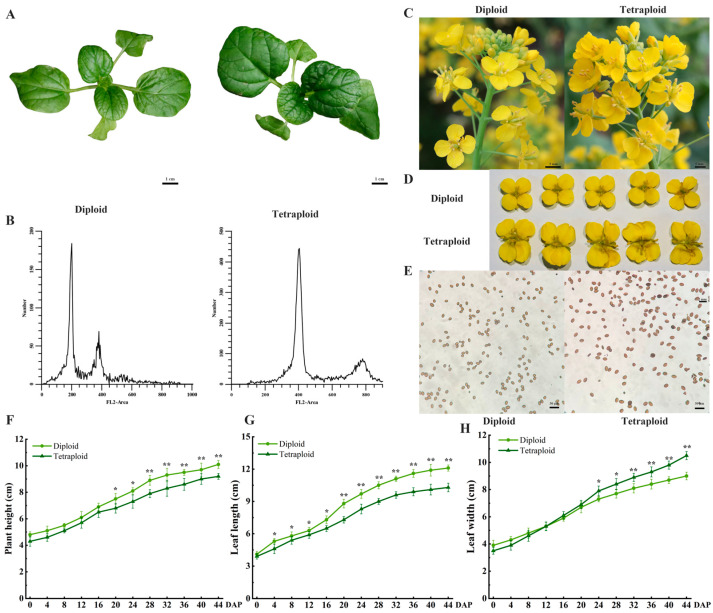
Ploidy analysis and phenotypes of diploid and tetraploid wucai. (**A**) Comparison of phenotypic characteristics among diploid and tetraploid wucai. (**B**) Ploidy level determined by flow cytometry. (**C**) Comparison of inflorescences between diploid and tetraploid wucai. (**D**) Comparison of complete flowers between diploid and tetraploid wucai. (**E**) Comparison of pollen grains between diploid and tetraploid wucai. (**F**) Plant height, (**G**) leaf length, and (**H**) leaf width of diploid and tetraploid wucai. Three groups of biological replicates were performed at each time point, and 15 plants were measured at each biological replicate. * represents significant difference (* *p* < 0.05); ** represents highly significant difference (** *p* < 0.01).

Significant morphological differences were observed between the diploid and tetraploid plants (Figure 2). Tetraploid wucai plants were larger with darker leaves (Figure 2A). Flow cytometry (FCM) analysis revealed that the peak value intensity of the tetraploid cells was approximately twice that of the diploid cells (Figure 2B). The flowers of the tetraploid wucai were significantly larger than those of the diploid wucai, while the pollen grains of the tetraploid wucai were enlarged and irregularly elliptical (Figure 2C–E). The fastest growth rate was observed between 24 and 40 days after planting (DAP) for both the diploid and tetraploid wucai. The tetraploid wucai plants were shorter and had smaller leaves than the diploid plants. Notably, the tetraploid wucai plants exhibited narrower leaf widths in the first 16 DAP, although their leaves gradually grew wider than those of the diploid plants after 20 DAP.

### 2.3. Anatomy and Microstructure of Tetraploid Wucai

**Figure 3:**
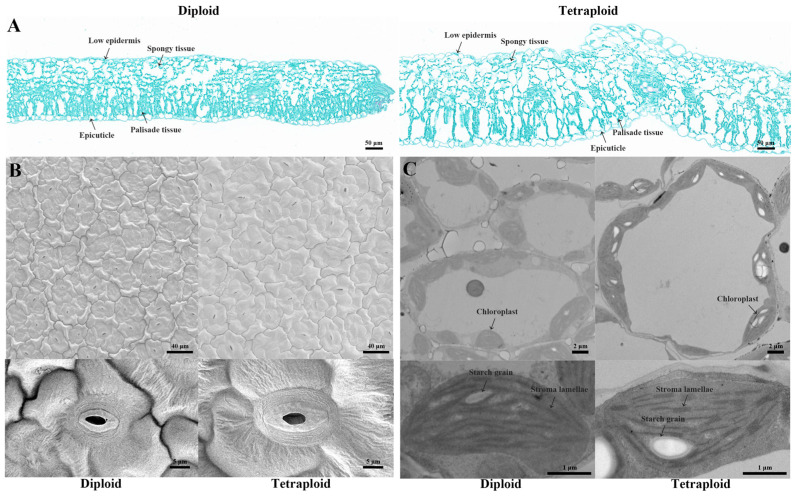
Anatomical and microstructural observations of diploid and tetraploid wucai. (**A**) Semi-thin slice of diploid and tetraploid wucai leaves. (**B**) Scanning electron microscopy observation of diploid and tetraploid wucai leaves. (**C**) Ultrastructure of chloroplasts of diploid and tetraploid wucai.

Leaf size, chloroplast morphology, and stomatal size are important indicators of plant development. Therefore, we further compared the microstructure of the diploid and tetraploid wucai (Figure 3). Overall, the tetraploid wucai had larger cells, thicker leaves, longer palisade tissue, and looser sponge tissue than the diploid wucai (Figure 3A). Scanning electron microscopy (SEM) revealed that, like most polyploid species, the tetraploid wucai exhibited lower stomatal density than the diploid wucai, although the tetraploid wucai had larger stomata (Figure 3B,C). Transmission electron microscopy (TEM) revealed that the chloroplasts of both the diploid and tetraploid wucai were structurally intact and well developed, with larger individual cells in the tetraploid, including a larger number of chloroplasts. In addition, larger starch granules were observed in the chloroplasts of the tetraploid plants, with thicker and more tightly arranged grana lamella, indicating that the photosynthetic capacity of the tetraploid plants may be stronger than that of the diploid plants.

### 2.4. Photosynthetic Differences between Diploid and Tetraploid Wucai

**Figure 4:**
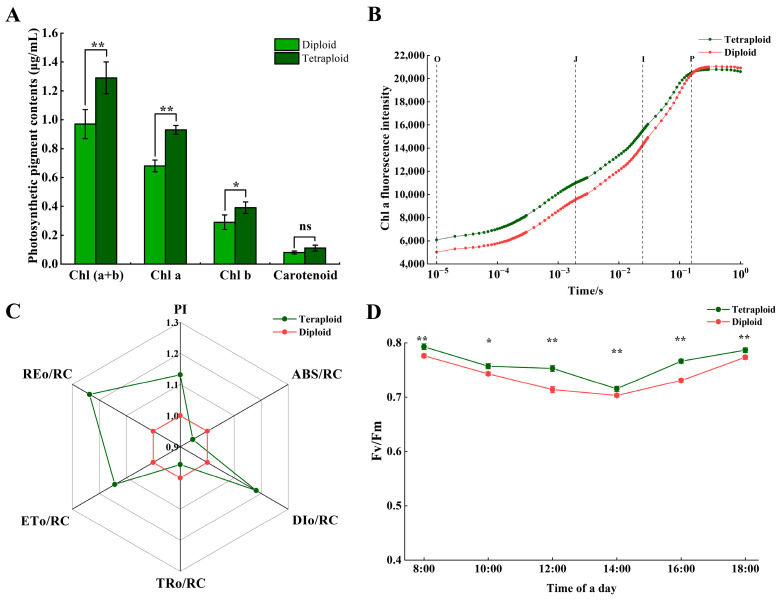
Comparison of photosynthetic performance between diploid and tetraploid wucai leaves. (**A**) Photosynthetic pigments content. (**B**) The fast chlorophyll a fluorescence transient plotted on a logarithmic time scale (0.00001–1 s). (**C**) Chlorophyll fluorescence kinetic parameters. PI: A performance index based on the absorption of light energy. ABS/RC: The amount of light absorbed per unit area (at t = 0). DIo/RC: Heat dissipation per unit area (at t = 0). TRo/RC: Capture of light energy per unit area (at t = 0). ETo/RC: Quantum yield of electron transfer per unit area (at t = 0). REo/RC: Electrons transferred to the end of PSI per unit area. (**D**) Diurnal variation curve of Fv/Fm. * represents significant difference (* *p* < 0.05); ** represents highly significant difference (** *p* < 0.01); ns represents no significant difference.

We further evaluated the differences in photosynthetic pigment content and the diurnal variation in photosynthetic parameters between the diploid and tetraploid wucai. The tetraploid wucai contained 36.76%, 34.48%, and 32.99% more chlorophyll a (Chla), chlorophyll b (Chlb), and total chlorophyll (total Chl) than the diploid plants, respectively (Figure 4A). Fast Chla fluorescence induction (OJIP transient) refers to the process of change from the lowest fluorescence O to the highest fluorescence P and reflects the activity of the primary photochemical reaction of photosystem II (PSII). The overall shapes and variation patterns of the OJIP curves were similar between the diploid and tetraploid wucai (Figure 4B). The RE_o_/RC, ET_o_/RC, and DI_o_/RC were 23.10%, 5.98% and 18.06% higher in tetraploid wucai leaves than in diploid leaves, respectively (Figure 4C). The diurnal Fv/Fm variation curves of both the diploid and tetraploid wucai leaves exhibited similar general trends (first decreasing and then increasing). However, the Fv/Fm of the tetraploid wucai was always higher than that of the diploid wucai throughout the day, and the tetraploid wucai recovered more quickly by 16:00 in the afternoon (Figure 4D). Together, these results suggest that the PSII reaction centers were more active in the tetraploid leaves, exhibiting higher electron transfer efficiency and stronger photosynthetic performance.

### 2.5. Comparison of Nutritional Quality between Diploid and Tetraploid Wucai

**Figure 5:**
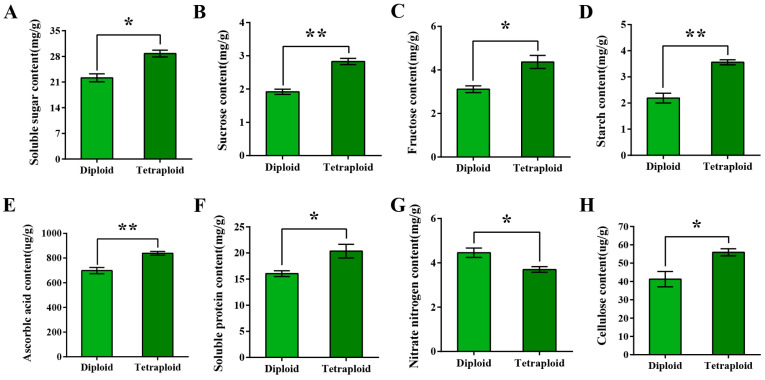
Comparison of the nutritional quality of diploid and tetraploid wucai leaves. (**A**) Soluble sugar content. (**B**) Sucrose content. (**C**) Fructose content. (**D**) Starch content. (**E**) Ascorbic acid content. (**F**) Soluble protein content. (**G**) Nitrate nitrogen content. (**H**) Cellulose content. * represents significant difference (* *p* < 0.05); ** represents highly significant difference (** *p* < 0.01).

We further measured the nutritional differences between the diploid and tetraploid wucai leaves. The tetraploid wucai leaves contained a significantly higher sugar content than the diploid leaves, including total soluble sugars, sucrose, fructose, and cellulose (Figure 5A–C,H). In addition, the contents of starch and soluble protein were also significantly higher in tetraploid plants (Figure 5D,F). Ascorbic acid (AsA) is an important antioxidant required for normal plant development and resistance to environmental stress. In addition, the optimal accumulation of AsA is closely related to redox homeostasis. The tetraploid wucai accumulated more AsA than the diploid wucai (Figure 5E). At low concentrations, nitrate nitrogen acts as signaling molecules rather than a nutrient to stimulate lateral root growth, while high concentrations inhibit plant growth. We found that the content of nitrate nitrogen in the tetraploid wucai leaves was significantly lower than in the diploid wucai leaves (Figure 5G). These results suggest that chromosomal duplication may result in the accumulation of more nutrients in the tetraploid wucai.

### 2.6. Transcriptomic Comparison between Diploid and Tetraploid Wucai at Different Developmental Stages

**Figure 6:**
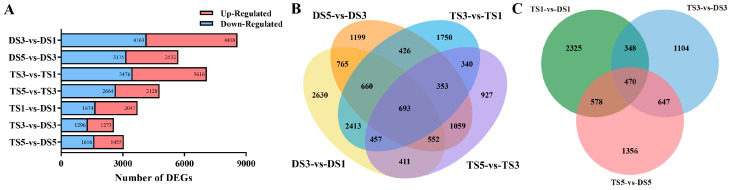
DEGs in diploid and tetraploid wucai. (**A**) Number of DEGs between diploid and tetraploid (DS5-vs-DS3, DS3-vs-DS1, TS5-vs-TS3, TS3-vs-TS1) and in tetraploid vs. diploid at different developmental stages (TS1-vs-DS1, TS3-vs-DS3, TS5-vs-DS5), as revealed by RNA-seq. (**B**) Venn diagrams of DEGs between the different pairwise comparisons (DS5-vs-DS3, DS3-vs-DS1, TS5-vs-TS3, TS3-vs-TS1). (**C**) Venn diagrams of DEGs between the different pairwise comparisons (TS1-vs-DS1, TS3-vs-DS3, TS5-vs-DS5).

For RNA-Seq analysis, pairwise comparisons of gene expression levels were performed using the aligned reads in order to identify the DEGs associated with the different developmental stages in the diploid and tetraploid plants (Supplementary Table S1). As shown in Figure 6, a total of 8581 DEGs (4163 upregulated and 4418 downregulated) were identified in DS3-vs-DS1, and 5707 DEGs (2532 upregulated and 3175 downregulated) were identified in DS5-vs-DS3. A total of 7092 DEGs were identified in TS3-vs-TS1, among which 3616 were upregulated and 3616 were downregulated. A total of 4832 DEGs were identified in TS5-vs-TS3, among which 2128 were upregulated and 2664 were downregulated, implying that tetraploidization resulted in fewer DEGs. In addition, a total of 3721 DEGs were identified in TS1-vs-DS1, of which 2047 were upregulated and 1674 were downregulated. Notably, the number of DEGs decreased in TS3-vs-DS3 (1273 upregulated and 1296 downregulated), while 3051 DEGs were identified in TS5-VS-DS5 (1435 upregulated and 1616 downregulated). A total of 693 DEGs were identified in the comparison group before and after the same ploidy development, while 470 DEGs were identified in the comparison group at the same period of a different ploidy.

Eight DEGs common to both the diploid and tetraploid wucai were selected for qRT-PCR verification (Supplementary Table S2). The results demonstrated a high degree of concordance between the qRT-PCR and RNA-Seq results, validating the reliability of the transcriptomic analysis for downstream investigations (Supplementary Figure S1).

### 2.7. Functional Analysis of Differentially Expressed Genes

**Figure 7:**
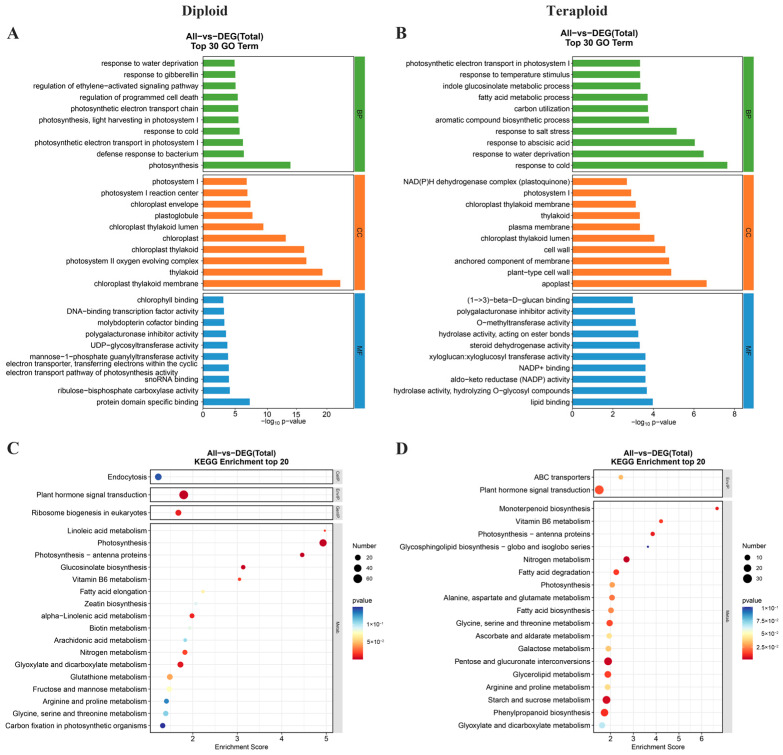
Comparative GO enrichment and KEGG pathway analysis of enriched DEGs. (**A**,**B**) Cellular component (CC), molecular function (MF) and biological process (BP) associated with DEGs based on pairwise comparisons of diploid (DS5-vs-DS3, DS3-vs-DS1) and tetraploid (TS5-vs-TS3, TS3-vs-TS1) plants, respectively. (**C**,**D**) The top 20 enriched pathways based on pairwise comparisons of diploid (DS5-vs-DS3, DS3-vs-DS1) and tetraploid (TS5-vs-TS3, TS3-vs-TS1) plants, respectively.

All DEGs identified in the diploid and tetraploid wucai at different developmental stages were annotated according to the following three Gene Ontology (GO) categories: cellular components (CC), molecular functions (MF), and biological processes (BP). DEGs identified in the diploid wucai were mainly enriched in the following BP terms: photosynthesis, defense response to bacterium, and photosynthetic electron transport in photosystem I; the following CC terms: chloroplast thylakoid membrane, thylakoid, and photosystem II oxygen evolving complex; and the following MF terms: protein domain-specific binding and lipid binding. Meanwhile, DEGs identified in tetraploid wucai were mainly enriched in the following BP terms: response to cold, response to water deprivation, and response to abscisic acid; the following CC terms: apoplast, plant-type cell wall, and anchored component of membrane; and the following MF terms: protein domain-specific binding and lipid binding (Figure 7A,B).

According to the Kyoto Encyclopedia of Genes and Genomes (KEGG) pathway analysis, DEGs identified in both the diploid and tetraploid wucai were mainly enriched in plant hormone signal transduction, photosynthesis, and vitamin B6 metabolism. Interestingly, DEGs identified in tetraploid wucai were also specifically enriched in starch and sucrose metabolism, pentose and glucuronate interconversions, and ascorbate and aldarate metabolism (Figure 7C,D).

### 2.8. Weighted Gene Co-Expression Network Analysis (WGCNA)

**Figure 8:**
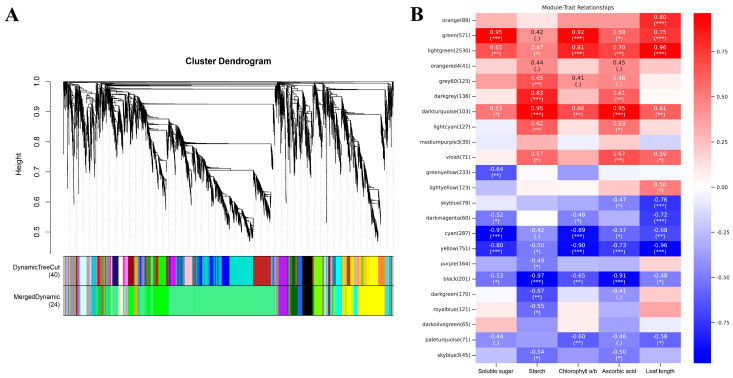
Weighted gene co-expression network analysis of diploid and tetraploid wucai. (**A**) Gene cluster tree and module division. (**B**) Correlation between modules and traits. * represents significant difference (* *p* < 0.05); ** and *** represent highly significant differences (** *p* < 0.01 and *** *p* < 0.001).

Hierarchical clustering was performed on the DEGs according to the gene expression patterns, and the minimum number of modules was set as 30. The DEG modules were further integrated according to the module characteristic values, and a total of 19 co-expressed modules were identified (Figure 8A). Different modules represent different gene expression patterns and are associated with different biological functions. In order to identify key modules related to differences in growth and development associated with ploidy, different modules associated with soluble sugars, starch, Chl a/b, and AsA were analyzed. Correlations between gene co-expression modules and different traits indicated that all phenotypic features were significantly correlated with at least one co-expressed module (Figure 8B).

**Figure 9:**
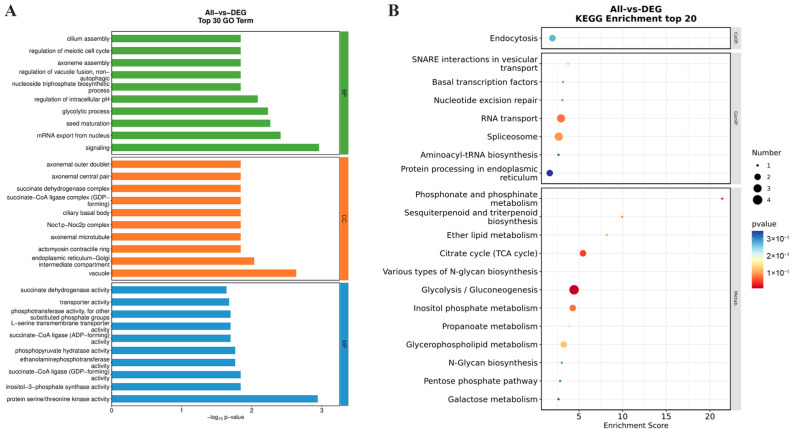
GO enrichment and KEGG pathway analysis of light green module genes. (**A**) GO enrichment. (**B**) KEGG pathway enrichment.

Through correlation analysis of modules and traits, it was found that the light green module was most relevant to the ploidy. According to GO enrichment analysis, genes in this module were involved in signaling, mRNA export from nucleus, vacuole, and protein serine/threonine kinase activity. In addition, genes in this module were enriched in the glycolysis/gluconeogenesis, TCA cycle, and RNA transport KEGG pathways, among others (Figure 9). These results suggest that glycolysis/gluconeogenesis and the TCA cycle may be key regulatory pathways underlying the differences between the diploid and tetraploid wucai.

### 2.9. Glycolysis Pathway Involved in Ploidy Development

**Figure 10:**
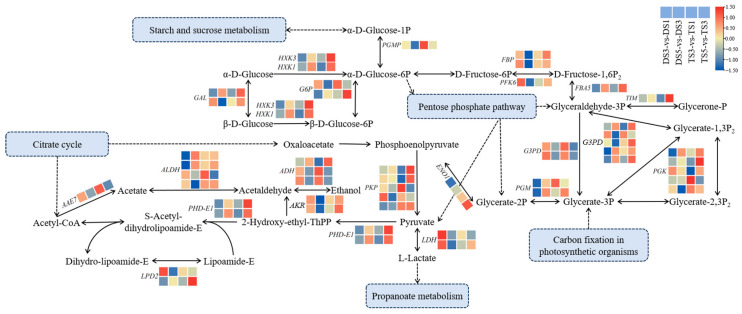
Glycolysis/gluconeogenesis pathway analysis. In the heatmap, blue genes are downregulated, while red genes are upregulated. Solid lines represent validated regulatory interactions, while dashed lines indicate predicted interactions. An arrow at the end of a line indicates positive regulation, while a line segment at the end of a line indicates negative regulation. The content of the pink dashed box represents the feedback loop.

Evaluation of the DEGs identified in the diploid and tetraploid wucai at different developmental stages revealed differential expression patterns of genes associated with the glycolysis pathway (Figure 10). For example, *HXK* (hexokinase) genes exhibited differential expressions at different developmental stages in the diploid and tetraploid wucai. In particular, *HXK3* was upregulated during the late developmental stage in the tetraploid wucai compared to the diploid wucai. In addition, *FBP* (fructose-1,6-bisphosphophase) genes were upregulated during the early developmental stage in both the diploid and tetraploid wucai but were downregulated in the diploid plants and upregulated in the tetraploid plants during the late developmental stage. Most *PKP* (pyruvate kinase) and *PGK* (phosphoglycerate kinase) genes were also significantly upregulated during the late developmental stage in the tetraploid wucai. Additionally, *LPD2* (dihydrolipoyl dehydrogenase 2) genes were also significantly activated during the later stages of development. The differential expression of these genes between the diploid and tetraploid wucai may affect plant growth and development.

## 3. Discussion

Ploidy breeding is increasingly seen as an effective method to develop ideal polyploid plants and create new germplasm [19]. Two methods are commonly employed to obtain polyploidy—natural generation and artificial induction. Most angiosperms exhibit a low natural mutation rate, and therefore artificial induction has become the favored method. Colchicine has been widely used as a low-cost but effective ploidy inducer. In this study, we treated seedling growth points with colchicine to induce autotetraploidy. In order to explore the rate of tetraploid induction, we applied colchicine at different concentrations and different numbers of treatments. The most effective mutagenic system was found to be six droplet treatments with 0.2% (w/v) colchicine applied to the inter-stem growth point, resulting in a doubling rate of 4.76%. However, different plant species respond differently to colchicine treatment. In peonies, the highest induction rate of diploid pollen was 47.39% when 0.4% colchicine was injected twice into flower buds [20]. Meanwhile, in cabbage, the highest induction rate was 8.6% when 0.15% colchicine was used to treat growth points six times [21]. Plants also exhibit morphological changes following colchicine induction. We observed that, following mutagenesis, the wucai leaves changed predominantly to become disc shaped. In fact, a vast majority of the tetraploid wucai leaves were disc shaped, which is of great significance for early rapid screening of suspected tetraploid plants in order to effectively reduce workload.

Increased ploidy can alter many traits and affect a variety of evolutionary and ecological processes [22]. WGD increases ploidy throughout the plant, resulting in an increase in cell size and biomass, as well as a characteristically “giant” phenotype [23]. Increasing ploidy also alters the contents of secondary metabolites. Compared with diploid energy willows, autotetraploid energy willows exhibit thicker stems, wider leaves, and larger cells. In addition, autotetraploid energy willows contain significantly higher contents of various phytohormones, resulting in increased net photosynthetic CO_2_ absorption [24]. Tetraploid oranges also exhibit similar phenotypic alterations [25]. In terms of plant height and root length, the growth rate of tetraploid wucai was significantly lower than that of diploid wucai. However, an increase in ploidy leads to enhanced photosynthesis and increased plant hormone content [26].

Stomata are channels on the surface of plant leaves [27] through which transpiration and carbon dioxide uptake are carried out, and they play a crucial role in supporting photosynthesis and water use efficiency [28]. Generally, stomata are more prominent in polyploid plants, although such plants also tend to exhibit decreased stomatal density [29]. Polyploid poplar exhibits improved water use efficiency and drought resistance, both of which are associated with increased stomatal volume and decreased stomatal density [30]. Here, we observed that tetraploid wucai exhibited slower growth but thicker, darker leaves and larger cells and organs (Figure 2). In addition, tetraploid wucai had higher photosynthetic efficiency and stronger photosynthetic performance, resulting in the synthesis of more photosynthetic substances (Figure 4). The palisade and sponge tissues of tetraploid wucai were thicker and arranged more tightly (Figure 3). We speculate that this may be related to the commonly observed enlargement of polyploid cells [31]. The increased stomatal volume and decreased stomatal density of tetraploid wucai may lead to increased transpiration rate and improved water use efficiency.

Both homologous and heterologous polyploidization can alter gene expression and gene networks [32]. In order to reveal the molecular mechanism of polyploidy-associated morphological variation, transcriptomic analysis was performed on diploid and tetraploid wucai plants. Polyploidy results in genome-wide gene duplication and genome redundancy, which may lead to gene silencing and loss of expression of duplicated genes [33]. This is crucial for maintaining stability in polyploid plants. We observed that the number of DEGs in the tetraploid wucai was significantly lower than in the diploid wucai at different developmental stages (Figure 6A). It could be inferred that polyploidization may maintain the normal growth and development of the tetraploid wucai through gene silence to repress the expression of some duplicate genes. In addition, we also observed fewer DEGs between the two ploidy wucai during the same developmental stage, which may be due to the fact that the growth and development of the diploid and tetraploid wucai were mostly regulated through common pathways, resulting in a decrease in the number of DEGs. Studies on the differential expression of transcription factor (TF)- and plant hormone-related genes in Chinese cabbages with different levels of ploidy have helped to elucidate the molecular basis of Chinese cabbage leaf head patterns [34]. DEGs in tetraploid *Dendrobium catenatum* are highly enriched in transport- and metabolism-related pathways, in which many *CesA*/*Csl*, *SWEET*, and *BGLU* genes are upregulated, suggesting that enhanced polysaccharide transport may be the key driver of increased polysaccharide content in tetraploid *D*. *catenatum* [35]. Polyploidization in *Isatidis Radix* regulates lignan biosynthesis and results in differential gene expression related to phenylpropanoid biosynthesis and plant hormone signal transduction. Furthermore, the lignin network contained 10 polyploidization-associated TFs and 17 fluctuating phenylpropanoid pathway complexes. Moreover, polyploidization increased the contents of active compounds in homologous tetraploid roots, and an analysis of the genes related to lignin biosynthesis revealed key functional and regulatory genes related to polyploidization [36]. Transcriptomic sequencing of the diploid and tetraploid wucai leaves at different developmental stages revealed that tetraploid wucai contained DEGs specifically enriched in the starch and sucrose metabolism, conversion of pentose and gluconate, and ascorbic acid and aldonate metabolism pathways (Figure 7).

Secondary metabolite biosynthesis is an important regulatory pathway for plant growth and development under homoploid conditions. WGCNA revealed the module most closely related to polyploidization, and the genes contained in this module were subjected to further enrichment analyses. Notably, the genes in this module were enriched in glycolysis/gluconeogenesis, TCA cycle, RNA transport, and other pathways (Figure 9). Glycolysis is a central metabolic pathway which provides energy and precursors for the synthesis of primary metabolites such as amino acids and fatty acids [37]. Glycolysis hydrolyses glucose into two three-carbon sugars, which are then further oxidized and converted into two pyruvate molecules. Interconversion of glucose-1-phosphate and glucose-6-phosphate by phosphoglucomutases (PGMP) connects the metabolism of these polysaccharides with central carbon metabolism [38]. We found that the expression of *PGMP* was upregulated in the tetraploid wucai but downregulated in the diploid wucai (Figure 10). Fructose 1,6-bisphosphatase (FBP) regulates plant growth in response to fructose signaling [39]. Interestingly, we found that *FBP* was upregulated in both diploid and tetraploid wucai during early development. However, *FBP* was downregulated in the diploid plants and upregulated in the tetraploid plants during late development. Phosphoglycerate kinase (PGK) not only converts 1,3-bisphosphoglycerate into 3-phosphoglycerate during glycolysis but also participates in the reverse reaction during gluconeogenesis and the Calvin–Benson cycle. PGKs are involved in not only glycolysis/gluconeogenesis but also in photosynthetic carbon metabolism [40]. Following tetraploidization, the majority of *PGKs* were upregulated, which may affect both sugar metabolism and photosynthesis in the tetraploid wucai [41]. The differential expression of glycolysis-associated genes may affect the growth and development of the tetraploid wucai, contributing to increased sugar accumulation and metabolic activity in the tetraploid wucai. The tetraploid wucai was also found to contain a greater Chl content than the diploid wucai, as well as enhanced photosynthesis [42]. Genes regulating amino acid transporters and chloroplast development have been observed to be significantly upregulated in other tetraploids [43]. The differential expression of these genes may improve photosynthetic efficiency and the accumulation of related nutrients in tetraploids [44].

## 4. Materials and Methods

### 4.1. Establishing Autotetraploid Wucai Lines

Wucai germplasm ‘W7-2’ (2n = 2x = 20) was provided by the Vegetable Genetic Breeding Laboratory of the College of Horticulture, Anhui Agricultural University, China. Seeds of diploid wucai ‘W7-2’ were sown in 50-cell trays. Following germination, fully expanded cotyledons were treated with colchicine droplets (10 μL) at their inter-stem growth points at 8:00 a.m. and 5:00 p.m., respectively. Three colchicine concentrations were tested (0.1%, 0.2%, 0.3% (w/v)), as well as different numbers of applications (2, 4, 6). A spot-drip distilled water treatment was used as the control group (CK). All experimental combinations are detailed in Table 2. The growth temperature was controlled at 25–27 °C during the day and 18–22 °C during the night.

### 4.2. Ploidy Analysis

Flow cytometry (FCM) was performed with a BD FACS Calibur flow cytometer (San Jose, CA, USA), according to the manufacturer’s instructions. Briefly, 0.2 g of fresh leaves were immersed in 1 mL of dissociation solution (4 mmol·L^−1^ MgCl_2_·6H_2_O, 5 mmol·L^−1^ HEPES, 1% (*v*/*v*) PVP-30, 0.5% (*v*/*v*) Triton-X, pH = 7.5). The leaves were then minced and filtered into a 50 mL centrifuge tube using a 40 μm filter. Following filtration, the suspension was employed for FCM. The diploid wucai leaves were used as a control.

### 4.3. Morphological and Microscopic Observation

Starting from the fourth true leaf, the phenotypes (leaf length, leaf width, plant height) of both the diploid and tetraploid wucai plants were observed every four days. Fifteen plants were randomly selected for measurement, with three biological replicates per plant. SEM was employed to observe leaf epidermis and stomata [45], and TEM was employed to observe chloroplast structure [46]. Longitudinal cross-sections of the paraffin-embedded leaves were prepared as described in Wang et al. [47] and observed with a light microscope.

### 4.4. Analysis of Photosynthetic Pigments and Chlorophyll Fluorescence Parameters

Total Chl was measured according to the method described in Arnon [48], with slight modifications. To extract total Chl, 0.2 g of fresh leaves were immersed in ethanol/acetone (1:1) in the dark for 24 h at approximately 4 °C. Subsequently, the absorbance at 665 and 649 nm was determined with a UV–vis spectrophotometer (TU1950, Beijing, China). The concentrations of Chla (CChla) and Chlb (CChlb) (μg·mL^–1^) were calculated as follows: CChla = 13.95 × A665 − 6.88 × A649; CChlb = 24.96 × A649 − 7.32 × A665. Chl fluorescence parameters were measured according to the method of Zhao [49].

### 4.5. Evaluation of Physiological Indicators

Five diploid and tetraploid wucai plants were randomly selected, cut into pieces, mixed, frozen in liquid nitrogen, and stored at −80 °C. Three technical replicates were used for each group. The anthrone colorimetric method was utilized to determine the contents of total soluble sugars, sucrose, fructose, and starch [50]. The soluble protein content was measured using the Coomassie Brilliant Blue method, with slight modifications [51]. The nitrate nitrogen content was measured according to Vickery’s method, with slight adjustments [52]. Commercial spectrophotometric assay kits were utilized to measure the contents of cellulose (BC4285; Solarbio, Beijing, China) and AsA (YFX0257; Solarbio, Beijing, China).

### 4.6. RNA Sequencing and Transcriptomic Analysis

For RNA-Seq analysis, diploid W7-2 (denoted as D) and autotetraploid W7-2 (denoted as T) wucai were studied at three development stages (4-week-old (S1), 10-week-old (S3), and 16-week-old (S5)). Total RNA was extracted using the TRIzol reagent (Invitrogen, USA), according to the manufacturer’s protocol. RNA purity and concentration were evaluated using the NanoDrop 2000 spectrophotometer (Waltham, MA, USA). RNA integrity was assessed using the Agilent 2100 Bioanalyzer (Santa Clara, CA, USA). RNA libraries were constructed using a VAHTS Universal V6 RNA-seq Library Prep Kit, according to the manufacturer’s instructions.

Transcriptomic sequencing and analysis were conducted by OE Biotech Co., Ltd. (Shanghai, China). RNA-Seq was performed on an Illumina Novaseq 6000 platform, generating 150 bp paired-end reads. Raw reads (fastq format) were processed using fastp to remove low-quality reads [53]. The resulting clean reads were mapped to the wucai genome using HISAT2 [54]. The fragments per kilo base of transcript per million mapped fragments (FPKM) [55] of each gene was calculated and the read count of each gene was obtained using HTSeq-count [56]. Principal component analysis (PCA) was performed using R (v3.2.0) to evaluate the biological duplication of samples.

Differential expression analysis was performed using DESeq2 [57]. Statistically significant DEGs were determined using Q-value < 0.05 and fold change (FC) > 2 or < 0.5 as thresholds. Hierarchical cluster analysis (HCA) of DEGs was performed using R (v3.2.0) to evaluate the expression patterns of genes in different groups and samples. A radar map of the top 30 genes was created to visualize the expression of up- or downregulated DEGs using the R package ggradar. Based on the hypergeometric distribution of the DEGs, R (v3.2.0) was used to perform GO [58], KEGG [59], Reactome, and WikiPathways enrichment analyses. Finally, R (v3.2.0) was used to create a column diagram, chord diagram, and bubble diagram of the significantly enriched terms.

### 4.7. Weighted Gene Co-Expression Network Analysis

A gene co-expression network was constructed using the WGCNA package for R, based on correlations between genes with similar expression trends across all samples. The WGCNA package converts adjacency relationships into topological overlaps, measures the network connectivity of genes, and generates networks. If the average FPKM value of a gene in 18 libraries exceeds 1, it is defined as a valid gene. The hierarchical clustering function was used to classify genes with similar expression profiles based on topological overlap measure (TOM) dissimilarity, with a minimum size of 30 in the gene tree diagram as the module. Modules were merged by calculating the differences between module feature genes.

### 4.8. Quantitative Real-Time PCR Validation

Diploid and tetraploid wucai leaves at different developmental stages were used for quantitative real-time PCR (qRT-PCR) validation. The experimental samples were consistent with the RNA-Seq samples. Specific primers were designed using PRIMER 6.0 (Supplementary Table S2). qRT-PCR was performed using a SYBR Premium Ex Taq II Kit (TaKaRa, Osaka Prefecture, Japan) for the Bio Rad CFX96 Real Time System (Hercules, CA, USA), according to the manufacturer’s instructions. The data were normalized using actin as an internal control. Three biological replicates were used for each sample and three technical replicates were used for each gene. The relative expression level was calculated using the 2^−ΔΔCt^ method [60].

## Figures and Tables

**Table 1:** Effect of colchicine treatment on doubling rate of wucai.

No.	Number of Individuals Treated	Number of Individuals Alive	Survival Rate	Number of Individuals Variable	Variable Rate	Number of Tetraploids	Doubling Rate
T1	189	175	92.59	161	85.19	2	1.06
T2	181	166	91.71	159	87.85	3	1.66
T3	182	160	87.91	161	88.46	4	2.2
T4	187	170	90.91	164	87.7	6	3.21
T5	186	162	87.1	169	90.86	7	3.76
T6	189	158	83.6	173	91.53	9	4.76
T7	195	173	88.72	179	91.79	5	2.56
T8	180	142	78.89	168	93.33	3	1.67
T9	184	136	73.91	172	93.48	1	0.54
Total	1673	1442	86.15	1506	90.02	40	2.38

**Table 2:** Experimental design of mutagenic effect of wucai treated with colchcines.

No.	Concentration of Colchicinne	Treatment Times
T1	0.1	2
T2	0.1	4
T3	0.1	6
T4	0.2	2
T5	0.2	4
T6	0.2	6
T7	0.3	2
T8	0.3	4
T9	0.3	6

## Data Availability

The raw RNA-Seq data used in this study have been deposited in the Nation Center for Biotechnology Information (NCBI) Sequence Read Archive (SRA) database under the accession number PRJNA1127318 (https://www.ncbi.nlm.nih.gov/sra/PRJNA1127318) accessed on 23 June 2024.

## Supplementary Materials

The following supporting information can be downloaded at: https://www.mdpi.com/article/10.3390/plants13162341/s1, Figure S1: qRT-PCR validation of DEGs in the diploid and tetraploid wucai at different development stages; Table S1: Summary of RNA-seq and alignment data for each sample; Table S2: Primer sequences of qRT-PCR.
