# Supplementary material for: Comparative Morphological, Physiological, and Transcriptomic Analyses of Diploid and Tetraploid Wucai (Brassica campestris L.)
Source: Plants (Basel). 2024 Aug 22;13(16):2341. doi: 10.3390/plants13162341 (PMC11359193; doi:10.3390/plants13162341)
Supplement: Supplementary file 1 [file plants-13-02341-s001.zip › plants-3097681-supplementary.pdf]

Comparative Morphological, Physiological, and Transcriptomic Analyses of Diploid and Tetraploid Wucai (*Brassica campestris* L.)

Supplementary Materials

Figure S1 qRT-PCR validation of DEGs in diploid and tetraploid wucai at different development stages.

Table S1 Summary of RNA-seq and alignment data for each sample.

Table S2 Primer sequences of qRT-PCR.

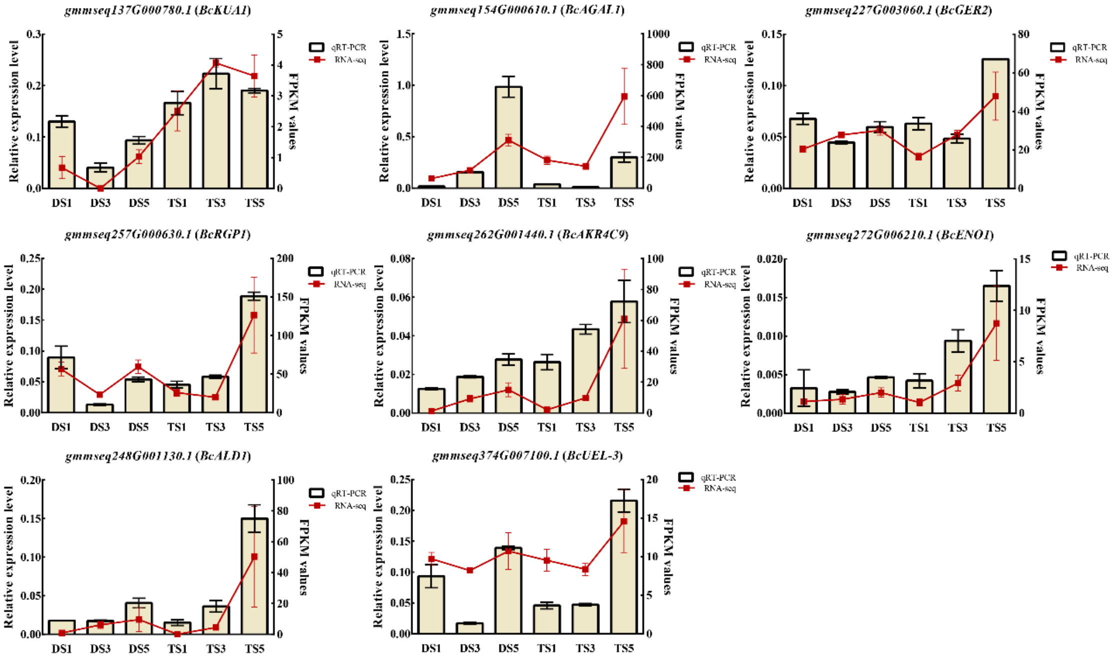

Figure S1 qRT-PCR validation of DEGs in diploid and tetraploid wucai at different development stages.

Table S1 Summary of RNA-seq and alignment data for each sample

Sample[a]	RawBases (G)	CleanBases (G)	ValidBases (%)[b]	GC (%)[c]	Total mapped reads (%)[d]
DS1-1	7.16	7.06	98.54	45.87	95.07
DS1-2	6.99	6.9	98.64	45.83	94.72
DS1-3	6.69	6.62	98.83	45.69	94.5
DS3-1	7.07	6.97	98.58	47.8	95.36
DS3-2	7.11	7.04	98.98	47.44	95.49
DS3-3	7.11	7.02	98.71	48.07	95.37
DS5-1	7.07	6.95	98.23	48.23	95.99
DS5-2	7.12	7.03	98.71	47.94	95.86
DS5-3	7.07	6.97	98.59	48.17	96.24
TS1-1	7.15	7.02	98.12	46.99	95.08
TS1-2	6.66	6.55	98.34	47.43	93.93
TS1-3	6.92	6.81	98.5	46.61	94.7
TS3-1	7.17	7.06	98.56	47.76	95.05
TS3-2	7.04	6.95	98.69	47.37	95.04
TS3-3	7.08	6.98	98.51	47.18	95.73
TS5-1	7.05	6.91	98.04	48.24	95.24
TS5-2	7.16	7.03	98.13	47.9	95.18
TS5-3	7.02	6.91	98.41	47.89	95.12

a The numbers 1,2 and 3 at the end of the sample name represent three replicates, respectively. D-diploid plants, T-tetraploid plants. S1, S3 and S5 represent three different developmental stages after plant;

b Percentage of bases with a Phred value of at least 30;

c Proportion of guanidine and cytosine nucleotides among total nucleotides;

d Percentage of clean reads mapped to the genome of Wucai.

Table S2 Primer sequences of qRT-PCR.

Primer sequences		
Gene name	Primer name	Primer sequences (5'-3')
BcActin	F	TGGGTTTGCTGGTGACGAT
	R	TGCCTAGGACGACCAACAATACT
gmmseq257G000630.1	F	CCTTACCATCTCATCATCGTCCA
	R	AGACCATGTAACCGAAGCAG
gmmseq262G001440.1	F	TGGCAACAGACTAAGCTACGA
	R	TTACCCATTTGAAGTCCCCAG
gmmseq272G006210.1	F	CTCAACGCTACTCTGAATCCG
	R	TCTACACTCCTTAGCCACCAC
gmmseq374G007100.1	F	CCCCAGTTTCACATCCCCAA
	R	TATATCCAGCGCCACCAGT
gmmseq248G001130.1	F	TTCGGCCTCATCTTTATCCTC
	R	CAAATTCACATTGCGGACCAG
gmmseq154G000610.1	F	TTCCCTTCTGGTATAAAAGCG
	R	ATAACTGGATACCTGACCGTTG
gmmseq227G003060.1	F	TCCGAACGATAATTTCCACCC
	R	TTCCTCAGCATCATTGGCCTT
gmmseq137G000780.1	F	AATCACAACGGTCACAACTCTC
	R	TCCCCATACTAGCACTTTTCC
